# Effect of chorioamnionitis on necrotizing enterocolitis in preterm infants: a multicenter cohort study

**DOI:** 10.3389/fped.2025.1620101

**Published:** 2025-07-04

**Authors:** Xin Guo, Jiaming Xian, Xianhong Chen, Meifen Li, Fengji Lin, Defei Ma, Rongrong Zhang, Guichao Zhong, Huiying Tu, Shujuan Zeng, Houxin Kang, Ya Pan, Xiaoli Li, Xueli Zhang, Zhangxing Wang, Hanni Lin, Shihua Yuan, Jing Han

**Affiliations:** ^1^ Department of Neonatology, Longgang District Maternity & Child Healthcare Hospital of Shenzhen City (Longgang Maternity and Child Institute of Shantou University Medical College), Shenzhen, Guangdong, China; ^2^ Shenzhen Clinical Medical College, Guangzhou University of Chinese Medicine, Shenzhen, Guang-dong, China; ^3^ Division of Neonatology, Longgang Central Hospital of Shenzhen, Shenzhen, Guangdong, China; ^4^ Department of Pediatrics, Longgang District Maternity & Child Healthcare Hospital of Shenzhen City (Longgang Maternity and Child Institute of Shantou University Medical College), Shenzhen, Guangdong, China; ^5^ Department of Neonatology, Shenzhen People’s Hospital, The Second Clinical Medical College of Jinan University, First Affiliated Hospital of Southern University of Science and Technology, Shenzhen, Guangdong, China; ^6^ Neonatal·Child Critical Child Health Care Division, The Central Hospital of Enshi Tujia and Miao Autonomous Prefecture, Enshi Tujia and Miao Autonomous Prefecture, Hubei, China; ^7^ Neonatal Department, Shenzhen Longhua Maternity and Child Healthcare Hospital, Shenzhen, Guangdong, China; ^8^ Division of Neonatology, Shenzhen Longhua People’s Hospital, Shenzhen, Guangdong, China; ^9^ Department of Neonatology, Shenzhen Luohu People’s Hospital, Shenzhen, Guangdong, China

**Keywords:** necrotizing enterocolitis, very low birth weight infants, chorioamnionitis, very preterm infants, death

## Abstract

**Background:**

To explore the association between maternal chorioamnionitis (CAM) exposure and necrotising enterocolitis (NEC) in very preterm infants (VPI) or very low birth weight (VLBWI).

**Methods:**

The aim of this multicentre cohort study was to investigate the impact of maternal CAM and its different staging on VPI or VLBWI NEC in six medical centres in Shenzhen between 2022 and 2023. The primary outcome was NEC (Bell staging ≥ II) and secondary outcomes included NEC or in-hospital mortality. Logistic regression adjusted for confounders identified through directed acyclic graphs (DAG) and literature review. The interaction effect of premature rupture of membranes was assessed using stratification and likelihood ratio tests.

**Results:**

In the cohort study, the prevalence of CAM was 44.31%, the prevalence of NEC was 5.38%, and the prevalence of NEC or death was 7.69%. Of the 288 participants whose mothers had been exposed to CAM, 1.04% had clinical CAM, 96.53% had histological CAM, and 2.43% were diagnosed with confirmed CAM. CAM was associated with NEC or death (aOR = 1.90, 95% CI 1.02–3.55); the confirmed CAM group showed a stronger association (aOR = 7.14, 95% CI 1.20–42.35). In preterm infants, CAM was significantly associated with NEC or death in cases of preterm membrane rupture (aOR = 2.18, 95% CI 1.07–4.44).

**Conclusions:**

There was a significant positive association between CAM and NEC or death in VLBWs or VPIs, which was mainly from the population with confirmed CAM. In premature rupture of membranes, the association between CAM and NEC or death was more significant.

## 1 Introduction

Necrotizing enterocolitis (NEC) is a leading cause of morbidity and mortality in neonatal intensive care units (NICU). The global incidence of NEC in all very low birth weight infants (VLBWI) admitted to the NICU is estimated to be 7% (1). Studies have shown that the average time of onset of NEC in very preterm infants (VPI) or VLBWI is in the third week of life (2). At the onset of NEC, symptoms such as feeding intolerance, meconium stools, and shock may be present. In severe cases, surgical intervention may be necessary, and in some cases, it can result in death. Children who undergo surgery for NEC may face long-term complications such as short bowel syndrome, intestinal stenosis, and neurodevelopmental disorders, even if they survive (3). Therefore, it is crucial to identify and prevent early risk factors for VPI or VLBWI to predict and prevent NEC. Currently, gestational diabetes mellitus, premature rupture of membranes, low birth weight, small for gestational age infants, sepsis, blood transfusion, congenital heart disease, respiratory distress syndrome, preterm delivery, and pneumonia have been linked to an increased risk of NEC (4). On the other hand, breastfeeding, probiotic use, prenatal glucocorticoid application, and hyperbilirubinemia may decrease this risk. Quality improvement initiatives, such as increasing breastfeeding rates, improving adherence to the feeding regimen, and preventing intestinal microbial aberrations, have been associated with a reduction in NEC (5). However, the role of chorioamnionitis (CAM) as a risk factor for NEC remains uncertain.

CAM, a common pregnancy complication, can occur before, during, or after delivery. When CAM is suspected or diagnosed, broad-spectrum antibiotics are typically administered to the mother according to protocols used in each country and continued until delivery (6). Acute CAM is a high-risk factor for early-onset sepsis, which increases the risk of early neonatal antibiotic exposure (7). The prevalence of CAM is related to gestational age at birth, with a higher prevalence in children born at younger gestational ages (8). CAM increases the incidence of preterm infants with both short- and long-term adverse outcomes, which has critical implications for child health (9, 10). A 2013 meta-analysis showed that clinical CAM and tissue-type CAM with fetal involvement were significantly associated with NEC in preterm infants, but the association between tissue-type CAM and NEC in preterm infants was not statistically significant (11). A study from Wenzhou, China, demonstrated that in preterm infants with preterm rupture of membranes and gestation of less than 34 weeks, those exposed to tissue-based CAM had a higher mortality rate, but it was not associated with the development of NEC (12). A national cohort study in the United States in 2024 also supported the association of maternal CAM with an increased incidence of NEC, but others have suggested the opposite (13, 14). Overall, the contribution of CAM to NEC in preterm infants remains controversial, and evidence from China is relatively scarce.

In this study, we aimed to investigate the impact of CAM on the incidence of NEC in VPI or VLBWI. The study was conducted as a cohort study in Shenzhen, China. In addition we will analyze the effect of different types of CAM on NEC, NEC or death. The results of this study may contribute to the understanding of the relationship between CAM and NEC in preterm infants. Furthermore, the findings may provide valuable evidence for implementing preventive measures for high-risk infants with CAM factors.

## 2 Methods

### 2.1 Study design

This study was a multicenter cohort study to determine whether CAM is associated with NEC in preterm infants. The study was approved by the Clinical Research Ethics Committee of Shenzhen People's Hospital (LL-KY-2022494-03). The cohort study was registered on the China Clinical Trial Registry (https://www.chictr.org.cn/) (ChiCTR2400090262). This study was conducted in accordance with Strengthening Reporting of Observational Studies in Epidemiology (STROBE) (see Supplementary File). Since the establishment of the Shenzhen Neonatal Data Network (SNDN) in 2022, homogeneous training has been provided to multiple obstetric and neonatal centers across the city (including the six centers in this study). SNDN-affiliated units have adopted uniform diagnostic criteria and collected clinical data using standardized case report forms.

### 2.2 Study population

VPI (gestational age <32 weeks) or VLBWI (birth weight <1,500 g) admitted to six medical centers (four general hospitals and two maternal and child health institutions) in Shenzhen between January 2022 and December 2023 were included in the study. The time period of the study included from the admission of the pregnant mothers to the obstetrics ward until the discharge of the preterm infants. During this period, maternal and neonatal medical record information was captured through the electronic medical record system. Participants whose mothers delivered at an outside hospital making it impossible to extract CAM information were excluded. The mean onset of NEC in very preterm/very low birth weight infants was the third week after birth. We therefore defined that neonates who did not observe NEC within 21 days of birth and who were discharged or transferred from the hospital for nonmedical reasons were excluded (2).

### 2.3 Definition of CAM

The presence or absence of CAM in the mother was obtained through the electronic medical record system. CAM is defined as follows (7, 15): (1) Histopathologically positive placenta with no clinical symptoms or only mild clinical symptoms is defined as histologic CAM. Histopathologically positive is defined as follows: early stage: mild chorioamnionitis, with neutrophils confined to the fibrous layer of the chorionic plate; intermediate stage: moderate chorioamnionitis, with neutrophil infiltration into the amnion and chorion; advanced stage: severe necrotizing chorioamnionitis, characterized by extensive neutrophil infiltration throughout the entire layers of the amnion and chorion, accompanied by necrosis; (2) Clinical CAM was defined as maternal fever (≥37.8°C) with at least two of the following criteria: maternal leukocytosis (leukocyte count >15,000/mm³), maternal tachycardia (heart rate ≥100 bpm), fetal tachycardia (heart rate ≥100 bpm), uterine tenderness, and foul-smelling vaginal discharge; (3) Confirmed CAM is defined as the presence of clinical manifestations that meet the above diagnostic criteria for clinical CAM, and positive placental histopathological examination results (with the same criteria as above).

### 2.4 Definition of outcomes

In our study, the presence of NEC during hospitalization was considered as the primary outcome, and the presence of NEC or death of preterm infants during hospitalization was considered as the secondary outcome. With reference to the Bell Modified Criteria, NEC was diagnosed at Bell stage ≥ II (16). Death was defined as a fatal event occurring in a preterm infant during hospitalization. Of the 650 participants ultimately included, there were no day-olds older than 31 days at the time of death. In this study, the composite outcome of NEC or death refers to infants diagnosed with NEC and all deaths, regardless of whether they had NEC.

### 2.5 Covariates

We selected as covariates factors that have been reported to be strongly associated with NEC (4), including: sex, gestational age, birth weight, prenatal use of cortisol, gestational diabetes mellitus, hypertensive disorders of pregnancy, multiple gestation, premature rupture of membranes, small for gestational age, hemoglobin minimum in the first 24 h of life, neonatal respiratory distress syndrome, neonatal pneumonia, full formula feeding, early-onset sepsis, late-onset sepsis, and patent ductus arteriosus. We also collected the length of hospital stay and hospitalization costs for preterm infants.

### 2.6 Statistical analysis

**Figure 1:**
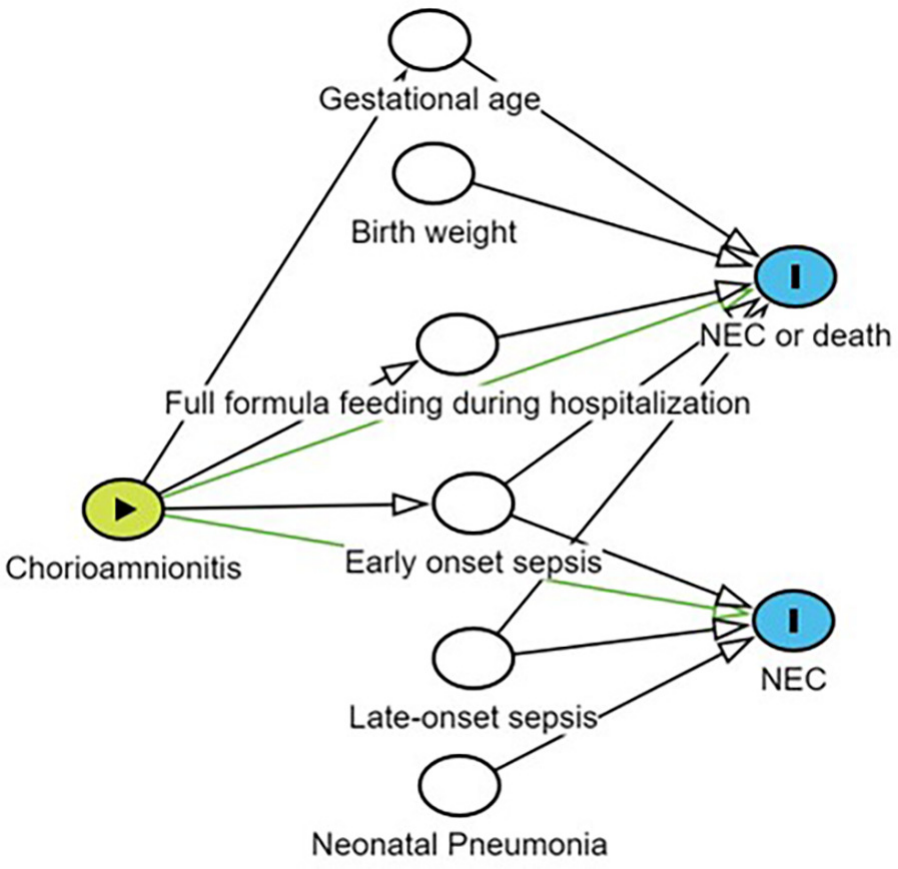
Potential confounders were identified by constructing a directed acyclic graph (DAG). Yellow nodes indicate exposure; Blank nodes indicate confounding factors related to the outcome that need to be adjusted; Blue nodes represent outcomes; Lines with arrows connecting two nodes indicate that there may be a correlation between the nodes; NEC, necrotizing enterocolitis.

Incidence of CAM and NEC were tabulated by covariates, and univariate associations were examined using chi-square tests or Fisher's exact tests (categorical data). Logistic regression was used to examine the effect of CAM on NEC, NEC or death. Potential confounders were identified by synthesizing the results of the literature research and statistically significant variables in univariate logistic regression (*P* < 0.1) by constructing directed acyclic graphs (DAGs) (Figure 1). In view of the fact that both gestational age at birth and premature rupture of membranes have been reported in the literature to affect outcomes and are correlated with exposures, we stratified by premature rupture of membranes and gestational age at birth, and validated the correlation between exposures and outcomes through interaction term modeling (4, 17). Interactions were tested using likelihood ratio tests. Estimated effects were expressed as odds ratios (OR) and adjusted odds ratios (aOR) with their 95% confidence intervals (95% CI). All analyses were performed using R software (version 4.3.3). *P*-values less than 0.05 were considered statistically significant.

## 3 Results

### 3.1 Overview of baseline characteristics of participants

**Figure 2:**
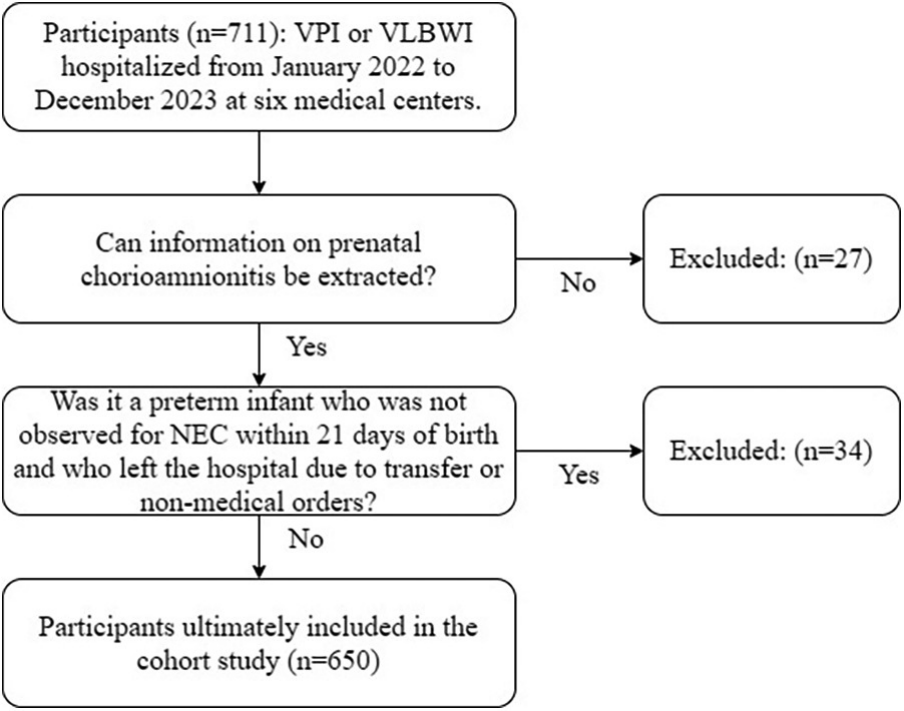
Flow chart for inclusion of populations in cohort studies. NEC, necrotizing enterocolitis; VLBWI, very low birth weight infants; VPI, very preterm infants.

A total of 711 participants were admitted to the six medical centers from January 2022 to December 2023, and 650 participants were ultimately enrolled in the cohort study (Figure 2). In the cohort study, the cumulative incidence of CAM was 44.31% (288/650), the cumulative incidence of NEC was 5.38% (35/650), and the cumulative incidence of NEC or death was 7.69% (50/650). Of the 288 participants whose mothers were exposed to CAM, 1.04% (3/288) had clinical CAM, 96.53% (278/288) had histologic CAM, and 2.43% (7/288) had confirmed CAM. As can be seen in Table 1, there were differences in the prevalence of CAM across medical centers, whereas the differences in the prevalence of NEC were not significant. CAM-exposed preterm infants were more likely to have a gestational age of less than 28 weeks, respiratory distress syndrome, early-onset sepsis, an increased risk of death, and a length of hospital stay of ≥46 days and a cost of ≥￥100 000 compared with non-CAM-exposed preterm infants. Among CAM-exposed preterm infants, the proportions of Small for gestational age and full formula feeding were lower. Among CAM-exposed preterm infants, there was a higher proportion of mothers with gestational diabetes and singleton pregnancies, and a lower proportion of mothers with gestational hypertension. We also know from Table 1 that the incidence of NEC was significantly higher in participants with the presence of late-onset sepsis than in those without late-onset sepsis. Participants with NEC had significantly higher rates of death, transfer or abandonment after 21 days of life, and hospitalization costs ≥￥100 000.

**Table 1:** Characteristics of participants in the cohort study and incidence of CAM and NEC (*n* = 650).

Characteristics	CAM		NEC	
	*N* (%)	*P*-value	*N* (%)	*P*-value
Total	288 (44.31)		35 (5.38)	
Medical centers		<0.001^a^		0.967^b^
Site 1	75 (40.76)		11 (5.98)	
Site 2	52 (78.79)		2 (3.03)	
Site 3	20 (25.00)		4 (5.00)	
Site 4	22 (27.85)		5 (6.33)	
Site 5	25 (36.76)		4 (5.88)	
Site 6	94 (54.34)		9 (5.20)	
Sex		0.691^a^		0.199^a^
Female	123 (45.22)		11 (4.04)	
Male	165 (43.65)		24 (6.35)	
Gestational age		0.004^a^		0.267^a^
<28 weeks	68 (56.20)		9 (7.44)	
≥28 weeks	220 (41.59)		26 (4.91)	
Birth weight		0.258^a^		0.129^a^
<1,000 grams	67 (48.55)		11 (7.97)	
≥1,000 grams	221 (43.16)		24 (4.69)	
Prenatal use of cortisol		0.056^a^		0.905^a^
No	20 (32.79)		4 (6.56)	
Yes	267 (45.56)		31 (5.29)	
Gestational diabetes		0.043^a^		0.605^a^
No	200 (41.93)		27 (5.66)	
Yes	88 (50.87)		8 (4.62)	
Hypertension during pregnancy		<0.001^a^		0.441^a^
No	244 (50.73)		24 (4.99)	
Yes	44 (26.19)		11 (6.55)	
Multiple births		0.003^a^		0.363^a^
No	228 (47.80)		28 (5.87)	
Yes	60 (34.68)		7 (4.05)	
Premature rupture of membranes		0.188^a^		0.569^a^
No	62 (39.74)		7 (4.49)	
Yes	226 (45.75)		28 (5.67)	
Small for gestational age		<0.001^a^		0.206^a^
No	269 (46.95)		28 (4.89)	
Yes	19 (24.68)		7 (9.09)	
Minimum hemoglobin value in the first 24 h of life		0.287^a^		0.812^a^
<130 g/L	37 (50.00)		3 (4.05)	
≥130 g/L	246 (43.46)		31 (5.48)	
Respiratory distress syndrome		<0.001^a^		0.120^a^
No	33 (27.27)		10 (8.26)	
Yes	255 (48.20)		25 (4.73)	
Neonatal Pneumonia		0.581^a^		0.075^a^
No	129 (43.14)		11 (3.68)	
Yes	159 (45.30)		24 (6.84)	
Early onset sepsis		0.011^a^		0.141^a^
No	239 (42.38)		27 (4.79)	
Yes	49 (56.98)		8 (9.30)	
Late-onset sepsis		0.111^a^		0.021^a^
No	259 (43.38)		28 (4.69)	
Yes	29 (54.72)		7 (13.21)	
Patent ductus arteriosus		0.855^a^		0.711^a^
No	130 (43.92)		17 (5.74)	
Yes	158 (44.63)		18 (5.08)	
Full formula feeding during hospitalization		<0.001^a^		0.664^a^
No	250 (48.08)		29 (5.58)	
Yes	38 (29.23)		6 (4.62)	
Discharge status		0.023^b^		0.024^b^
Survived	272 (43.45)		31 (4.95)	
Dead	13 (76.47)		2 (11.76)	
Discharged from hospital after 21 days after birth without medical advice	3 (42.86)		2 (28.57)	
Length of hospitalization		0.001^a^		0.519^a^
<46 days	118 (37.70)		15 (4.79)	
≥46 days	170 (50.45)		20 (5.93)	
Hospitalization costs		<0.001^a^		0.026^a^
<￥100 000	159 (38.59)		16 (3.88)	
≥￥100 000	129 (54.20)		19 (7.98)	

^a^ Chi-square test.

^b^ Fisher exact.

### 3.2 Effect of CAM on NEC

As can be seen in Table 2, the incidence of NEC was not significantly different between preterm infants exposed to CAM and those not exposed to CAM. The incidence of NEC did not differ significantly between preterm infants unexposed to CAM, exposed to clinical CAM, histologic CAM, and confirmed CAM. The univariate analysis revealed that CAM (OR = 1.35, 95% CI 0.68–2.68), histologic CAM (OR = 1.32, 95% CI 0.66–2.64), and confirmed CAM (OR = 3.38, 95% CI 0.39–29.69) each exhibited non-significant positive associations with elevated odds of NEC. None of these associations reached statistical significance.

**Table 2:** Effect of CAM on the presence of NEC on VPI or VLBWI (*n* = 650).

Exposures	N (%)	NEC		Unadjusted OR (95% CI), *P*-value	Adjusted OR (95% CI)^a^, *P*-value	Adjusted OR (95% CI)^b^, *P*-value
		N (%)	*P*-value			
CAM			0.383^c^			
No	362 (55.69)	17 (4.70)		1.00 (Reference)	1.00 (Reference)	1.00 (Reference)
Yes	288 (44.31)	18 (6.25)		1.35 (0.68–2.68), *P* = 0.385	1.35 (0.68–2.69), *P* = 0.392	1.23 (0.61–2.47), *P* = 0.564
Types of CAM			0.372^d^			
No	362 (55.69)	17 (4.70)		1.00 (Reference)	1.00 (Reference)	1.00 (Reference)
Clinical CAM	3 (0.46)	0 (0.00)		e	e	e
Histogenic CAM	278 (42.77)	17 (6.12)		1.32 (0.66–2.64), *P* = 0.429	1.31 (0.65–2.64), *P* = 0.447	1.19 (0.59–2.42), *P* = 0.624
Confirmed CAM	7 (1.08)	1 (14.29)		3.38 (0.39–29.69), *P* = 0.272	3.94 (0.44–35.25), *P* = 0.220	3.26 (0.34–30.87), *P* = 0.303

^a^ Adjusted covariates included sex, gestational age, and birth weight.

^b^ Adjusted for sex, birth weight, gestational age, and all confounders in the DAGs: neonatal pneumonia, early-onset sepsis, and late-onset sepsis.

^c^ Chi-square test.

^d^ Fisher exact.

^e^ Due to the small number of cases, convergence has not been achieved.

In a multifactorial logistic regression model, after correcting for covariates such as sex, gestational age, and birth weight, CAM was associated with NEC, but this association did not reach statistical significance (aOR = 1.35, 95% CI 0.68–2.69). Histologic CAM (aOR = 1.31, 95% CI 0.65–2.64) and confirmed CAM (aOR = 3.94, 95% CI 0.44–35.25) were also associated with NEC, but this association did not reach statistical significance.

After adjusting by constructing DAGs to identify potential confounders by further incorporating sex, gestational age, birth weight, neonatal pneumonia, early-onset sepsis, and late-onset sepsis, CAM still showed an association with NEC (aOR = 1.23, 95% CI 0.61–2.47), but this association did not reach statistical significance. Histologic CAM (aOR = 1.19, 95% CI 0.59–2.42) and confirmed CAM (aOR = 3.26, 95% CI 0.34–30.87) still exhibited an association with NEC, but this association did not reach statistical significance.

### 3.3 Effect of CAM on NEC or death

As can be seen in Table 3, the incidence of NEC or death was higher in preterm infants exposed to CAM compared to those not exposed to CAM. The incidence of NEC or death was significantly different between preterm infants not exposed to CAM, those exposed to clinical CAM, histologic CAM, and those with confirmed CAM. The results of univariate analysis showed that CAM was associated with NEC or death (OR = 1.82, 95% CI 1.01–3.26) with statistical significance. Among the different subtypes of CAM, confirmed CAM was associated with NEC or death (OR = 6.50, 95% CI 1.19–35.48) and was statistically significant.

**Table 3:** Effect of CAM on the composite outcome of NEC or death in VPI or VLBWI (*n* = 650).

Exposures	N (%)	NEC or death		Unadjusted OR (95% CI), *P*-value	Adjusted OR (95% CI)^a^, *P*-value	Adjusted OR (95% CI)^b^, *P*-value
		N (%)	*P*			
CAM			0.042^c^			
No	362 (55.69)	21 (5.80)		1.00 (Reference)	1.00 (Reference)	1.00 (Reference)
Yes	288 (44.31)	29 (10.07)		1.82 (1.01–3.26), *P* = 0.045	1.71 (0.94–3.11), *P* = 0.077	1.90 (1.02–3.55), *P* = 0.044
Types of CAM			0.017^d^			
No	362 (55.69)	21 (5.80)		1.00 (Reference)	1.00 (Reference)	1.00 (Reference)
Clinical CAM	3 (0.46)	1 (33.33)		8.12 (0.71–93.20), *P* = 0.093	4.93 (0.38–63.49), *P* = 0.221	4.53 (0.32–64.57), *P* = 0.265
Histogenic CAM	278 (42.77)	26 (9.35)		1.68 (0.92–3.05), *P* = 0.091	1.59 (0.86–2.92), *P* = 0.138	1.76 (0.93–3.33), *P* = 0.080
Confirmed CAM	7 (1.08)	2 (28.57)		6.50 (1.19–35.48), *P* = 0.031	7.79 (1.36–44.62), *P* = 0.021	7.14 (1.20–42.35), *P* = 0.030

^a^ Adjusted covariates included sex, gestational age, and birth weight.

^b^ Adjusted for sex, and all confounders in the DAGs: birth weight, gestational age, full formula feeding during hospitalization, early-onset sepsis, and late-onset sepsis.

^c^ Chi-square test.

^d^ Fisher exact.

In a multifactorial logistic regression model, after correcting for covariates such as sex, gestational age, and birth weight, CAM was associated with NEC or death, but this association did not reach statistical significance (aOR = 1.71, 95% CI 0.94–3.11). Among the different subtypes of CAM, confirmed CAM was associated with NEC or death (aOR = 7.79, 95% CI 1.36–44.62) with statistical significance.

After adjusting by constructing DAGs to identify potential confounders by further incorporating sex, gestational age, birth weight, full formula feeding during hospitalization, early-onset sepsis, and late-onset sepsis, CAM still exhibited an association with NEC or death (aOR = 1.90, 95% CI 1.02–3.55), and this association was statistically significant. Confirmed CAM still exhibited an association with NEC or death (aOR = 7.14, 95% CI 1.20–42.35), and this association was statistically significant.

### 3.4 Impact of stratification of premature rupture of membranes on outcomes

Stratification according to preterm rupture of membranes revealed that CAM was more strongly associated with NEC or death in preterm infants with preterm rupture of membranes exposure, but the interaction between groups did not reach statistical significance (Table 4). In stratified analyses, CAM was significantly associated with NEC or death in preterm infants with preterm rupture of membranes exposure (aOR = 2.18, 95% CI 1.07–4.44), whereas there was no significant association in preterm infants without preterm rupture of membranes exposure (aOR = 0.98, 95% CI 0.23–4.18). In stratified analyses, CAM as well was not associated with NEC.

**Table 4:** Effects and interactions of CAM on NEC or death composite outcome, NEC, as assessed by stratification of gestational age and premature rupture of membranes.

Subgroup	NEC				NEC or death			
	OR (95% CI), *P*-value	*P*-value for interaction	Adjusted OR (95% CI)^a^, *P*-value	*P*-value for interaction	OR (95% CI), *P*-value	*P*-value for interaction	Adjusted OR (95% CI)^b^, *P*-value	*P*-value for interaction
Gestational age		0.632		0.694		0.355		0.257
<28 weeks	0.97 (0.25–3.81), 0.968		0.85 (0.19–3.73), 0.829		2.49 (0.83–7.42), 0.102		5.27 (1.28–21.62), 0.021	
≥28 weeks	1.43 (0.65–3.15), 0.374		1.34 (0.60–3.00), 0.478		1.34 (0.65–2.77), 0.430		1.33 (0.63–2.83), 0.453	
Premature rupture of membranes		0.821		0.819		0.543		0.527
No	1.14 (0.25–5.30), 0.863		0.95 (0.19–4.74), 0.949^c^		1.23 (0.32–4.76), 0.767		0.98 (0.23–4.18), 0.982^d^	
Yes	1.39 (0.65–3.00), 0.394		1.26 (0.57–2.78), 0.573^c^		1.96 (1.02–3.77), 0.044		2.18 (1.07–4.44), 0.033^d^	

^a^ Adjusted for sex, birth weight, and all confounders in the DAGs: neonatal pneumonia, early-onset sepsis, and late-onset sepsis.

^b^ Adjusted for sex, and all confounders in the DAGs: birth weight, full formula feeding during hospitalization, early-onset sepsis, and late-onset sepsis.

^c^ Adjusted for sex, birth weight, gestational age, and all confounders in the DAGs: neonatal pneumonia, early-onset sepsis, and late-onset sepsis.

^d^ Adjusted for sex, and all confounders in the DAGs: birth weight, gestational age, full formula feeding during hospitalization, early-onset sepsis, and late-onset sepsis.

For the composite outcome of NEC or death, the unadjusted OR across different gestational age strata did not reach statistical significance, but the aOR in infants born at gestational age <28 weeks was statistically significant (OR=5.27, 95% CI 1.28–21.62). In stratified analyses, CAM was also not associated with NEC. It indicates that the interaction effect of gestational age on the correlation between CAM and “NEC or death” was not significant.

## 4 Discussion

Evidence from China on the relationship between CAM and NEC remains scarce. A single-center study in Wenzhou, China, found that in preterm infants less than 34 weeks of gestational age exposed to preterm rupture of membranes, histologic CAM was not associated with the development of NEC (12). The present study is a multicenter study to determine whether maternal exposure to CAM is associated with the development of NEC in VPI or VLBWI and has a larger sample size than the study in Wenzhou published in 2015. This study is a multicenter study to determine the association of maternal CAM exposure with NEC in preterm infants. In VLBWI or VPI, our finding was that maternal CAM exposure may not be associated with NEC. Given the competing risks of mortality and NEC in VLBWI or VPI, we also validated the composite outcome of NEC or mortality. The results suggest that there may be a significant association between maternal exposure to CAM and increased rates of NEC or death, and that this association may originate from the group with confirmed CAM. The results of the stratified analysis may support a stronger association between CAM and NEC or death in those exposed to preterm premature rupture of membranes.

Consistent with our findings, in a national cohort in the United States, this lack of association between CAM exposure and NEC was also observed in mothers of VLBWI (14). The research team also found that CAM was positively correlated with the incidence of NEC in mature newborns, but not in VPI. Therefore, they hypothesized that there are differences in risk factors associated with necrotizing enterocolitis (NEC) between very preterm and more mature newborns. However, this study did not discuss the association of different types of CAM with NEC, nor did it consider the composite outcome of NEC or death. The systematic review published in 2013 was a long time ago, so the results of more recent studies need to be synthesized to clarify the relationship between CAM and NEC from multiple perspectives (11). Furthermore, large multicenter cohort studies outside of the United States are needed to validate these findings.

It is also important to consider the competing risk between death and NEC outcomes in VPI and VLBWI, making it necessary to study the combined outcomes of NEC or death. To our knowledge, no studies have examined the association between maternal CAM exposure and the combined outcome of NEC or death, and our study aims to fill this gap. As the incidence of NEC or death has decreased over time, likely due to advancements in medical technology and increased healthcare awareness (18). it is crucial to identify prenatal risk factors for NEC or death in order to prevent these outcomes. Previous research has shown that prenatal steroid use can reduce the incidence of NEC or death in VPIs born to mothers with hypertensive disorders of pregnancy (19). Therefore, early detection of prenatal factors contributing to NEC or death is crucial for prenatal prevention. Our study is the first to conclusively demonstrate that maternal CAM exposure is one of the important prenatal high-risk factors for the combined outcome of NEC or death. Additionally, CAM is a known risk factor for early-onset sepsis and is often an indication for early empirical antibiotic use. However, previous studies have found that prolonged initial empiric antibiotic therapy (≥ 5 days) is associated with a combined outcome of late-onset sepsis, NEC, or death after 7 days of life (20). Therefore, it is reasonable to hypothesize that CAM may contribute to the outcome of NEC or death by influencing clinical antibiotic practice, but further studies are needed to validate this conclusion. In high-risk preterm infants with chorioamnionitis, implementing appropriate feeding regimens and promoting breastfeeding can play a protective role in reducing the incidence of NEC or death (21, 22).

Consistent with our results, a Korean study in 2017 also concluded that neither clinical nor histologic CAM was a risk factor for NEC (13). However a study published in 2019 also agrees that clinical and histologic CAM are risk factors for NEC (23). A meta-analysis published in 2013 concluded that clinical CAM and fetal involvement of histologic CAM were significantly associated with NEC in preterm infants (11). However, the meta-analysis did not explore the association of preterm infants who met both clinical CAM and histologic CAM with NEC, and NEC or death. The concept of confirmed CAM is more commonly used in Chinese neonatal clinical practice, which implies more severe CAM. We provide evidence for the first time that confirmed CAM may be significantly associated with NEC or mortality, whereas histological or clinical CAM alone did not show such an association. However, the small number of confirmed CAM and clinical CAM cases cannot be ruled out as a factor influencing the results. This suggests that neonatologists should follow up on the results of placental pathology after finding that the mother of a VPI or VLBWI meets clinical CAM criteria.

Since the results regarding the relationship between CAM and NEC in the group of preterm infants with preterm rupture of membranes are not consistent, we conducted a stratified analysis based on the presence or absence of preterm rupture of membranes (12, 17, 24). Premature rupture of membranes and CAM are at the same high risk for early-onset sepsis and are strongly associated with initial empiric antibiotic exposure. Our findings also suggest that CAM may be associated with a high risk of NEC or mortality in preterm infants with preterm premature rupture of membranes. Data from the United States suggest that preterm premature rupture of membranes leads to CAM, NEC, and death, and that prenatal antibiotic use in women with preterm premature rupture of membranes reduces the incidence of CAM and NEC (17). In pregnancy cohorts with histologic CAM, the presence of a fetal inflammatory response was associated with clinical CAM, premature rupture of membranes in preterm infants, prenatal use of steroids and antibiotics, and adverse neonatal outcomes (25). This suggests that CAM may be an important intermediate factor between premature rupture of membranes and NEC or death in VLBW or VPI, whereas the fetal inflammatory response may be a common pathogenesis for all three of the above (25, 26). Of course further clinical observations and animal experiments are needed to verify this. As previously reported in the literature, the association between chorioamnionitis (CAM) and necrotizing enterocolitis (NEC) may not be significant in preterm infants with lower birth weights or smaller gestational ages, which is consistent with the results of our stratified analysis by gestational age (14). However, in preterm infants with gestational age <28 weeks, after adjusting for sex, birth weight, full formula feeding during hospitalization, early-onset sepsis, and late-onset sepsis, we found an association between CAM and NEC or death. This may be attributed to the higher grade of histological CAM in younger gestational ages, reflecting weaker tissue-specific immune competence of gestational tissues and more severe fetal inflammatory response in preterm infants (27). However, limited by the small sample size in the gestational age <28 weeks group and the influence of other potential confounding factors, this finding should be interpreted with caution in the absence of corroboration from larger sample size data.

This study has the following advantages. This study is the first to find that CAM is associated with a composite outcome of confirmed CAM and NEC or death. This study is the first multicenter study in China to investigate the relationship between CAM and NEC, NEC, or death, and currently has the largest sample size in this field in China. Future studies can be based on the results of this study to provide guidance for the management of CAM in pregnant women and the prevention and treatment of NEC. However, there are some limitations to this study. Some cases of infants whose mothers had chorioamnionitis may have been missed due to incomplete prenatal information on discharge from outside hospitals. However, this limitation was compensated for by the large number of cases included. This paper is a retrospective study, and data collection relied mainly on electronic medical records, which did not allow information to be obtained on whether chorioamnionitis was acute or chronic. The incidence of surgical NEC was low and therefore not isolated as a secondary outcome. Additionally, in the subgroup analysis, only three clinical CAM cases and seven confirmed CAM cases were included, which may have caused the results to be influenced by outliers, limiting the robustness and generalizability of the findings. A larger sample size will be required in the future to validate the conclusions of the subgroup analysis. Our study adjusted for covariates related to NEC, but did not assess the effects of early feeding strategies and antibiotics on CAM, NEC or death. Therefore, future studies should analyze these factors and the interaction between CAM and NEC to reinforce the conclusions of our study. Concerns about prognosis and economic burden are the primary factors contributing to non-medical discharge abandonment. These factors may introduce selection bias. Additionally, setting a 21-day postnatal exclusion criterion may introduce selection bias, leading to preterm infants discharged after 21 days due to transfer or non-medical reasons being categorized into the negative outcome group if they develop NEC or mortality. Therefore, caution is required when adopting this timeframe as an inclusion-exclusion criterion in future studies, and more data on the onset timing of NEC in VPI or very low birth weight infants VLBWI are needed to support its validity.

## 5 Conclusion

Our cohort found no association between maternal CAM exposure and NEC in VLBWIs or VPIs. However, there was a significant association between maternal CAM exposure and increased incidence of NEC or death in VLBWIs or VPIs, and this association was predominantly from the population with confirmed CAM. We also found a stronger association between CAM and NEC or death in VLBWIs or VPIs with premature rupture of membranes.

## Data Availability

The raw data supporting the conclusions of this article will be made available by the authors, without undue reservation.
